# Evidence for host genetic regulation of altered lipid metabolism in experimental toxoplasmosis supported with gene data mining results

**DOI:** 10.1371/journal.pone.0176700

**Published:** 2017-05-01

**Authors:** Ivan Milovanović, Miloš Busarčević, Alexander Trbovich, Vladimir Ivović, Aleksandra Uzelac, Olgica Djurković-Djaković

**Affiliations:** 1 Institute for Pathologic Physiology, School of Medicine, University of Belgrade, Belgrade, Serbia; 2 National Reference Laboratory for Toxoplasmosis, Center of Excellence for Food- and Vector-borne Zoonoses, Institute for Medical Research, University of Belgrade, Belgrade, Serbia; Universita degli Studi di Bologna, ITALY

## Abstract

*Toxoplasma gondii* is one of the most successful parasites on Earth, infecting a wide array of mammals including one third of the global human population. The obligate intracellular protozoon is not capable of synthesizing cholesterol (Chl), and thus depends on uptake of host Chl for its own development. To explore the genetic regulation of previously observed lipid metabolism alterations during acute murine *T*. *gondii* infection, we here assessed total Chl and its fractions in serum and selected tissues at the pathophysiological and molecular level, and integrated the observed gene expression of selected molecules relevant for Chl metabolism, including its biosynthetic and export KEGG pathways, with the results of published transcriptomes obtained in similar murine models of *T*. *gondii* infection. The serum lipid status as well as the transcript levels of relevant genes in the brain and the liver were assessed in experimental models of acute and chronic toxoplasmosis in wild-type mice. The results showed that acute infection was associated with a decrease in Chl content in both the liver and periphery (brain, peripheral lymphocytes), and a decrease in Chl reverse transport. In contrast, in chronic infection, a return to normal levels of Chl metabolism has been noted. These changes corresponded to the brain and liver gene expression results as well as to data obtained via mining. We propose that the observed changes in Chl metabolism are part of the host defense response. Further insight into the lipid metabolism in *T*. *gondii* infection may provide novel targets for therapeutic agents.

## Introduction

*Toxoplasma gondii* is considered one of the most successful parasites on Earth due to its omnipresence and widest array of hosts, including all mammals. The genus comprises a single species infective for all hosts, with limited genetic diversity in Europe and North America where all isolates belong to three clonal genotypes (type I, II and III). However, a wider genetic diversity characterized by non-clonal, atypical strains is found in South America and Africa, and is thought to be related to the presence of diverse *Felidae* as the only definitive host in which sexual reproduction, and consequentially, genetic recombinations, occur. In intermediate hosts, *T*. *gondii* occurs in two forms, the metabolically active rapidly proliferating tachyzoite which characterizes acute infection and the presumably metabolically inert encysted bradyzoite, characteristic of chronic infection; the parasite readily converts between the two in response to the hospitality or hostility of the host environment (mostly depending on the immune response) but is never eliminated from the infected host. The encysted bradyzoites generally localize in the host brain and muscles and remain latent under the control of the host adaptive immune response.

Human infection is widespread; it has been estimated that one third of the global population is infected [1,2]. However, infection is generally mild and self-limiting, except in population categories with an incompetent immune system, including the unborn baby and immunosuppressed individuals, in whom it may cause life-threatening disease [2]. Treatment options have not much advanced for decades and there is still no drug able to eliminate encysted parasites. Therefore, there is an urgent need for new drugs.

A potential novel drug target may be Chl metabolism [3]. It has been known for some time that *T*. *gondii* is not capable of synthesizing cholesterol (Chl), and thus depends on uptake of host Chl for its own development [4]. Chl metabolism in mammalian cells involves direct transport of preformed sterol from the liver in the form of very low density lipoprotein (VLDL) and low density lipoprotein (LDL) to the periphery. In fact, it has been shown that *T*. *gondii* acquires host Chl via endocytosis mediated by the LDL receptor [3, 5] or the LDL receptor-related protein [6]. On the other hand, tissue Chl is transported from peripheral cells through the plasma compartment back to the liver in the form of high density lipoprotein (HDL) [7].

Although Chl apparently has an important role in the pathogenesis of toxoplasmosis, this topic has, interestingly enough, not received sufficient attention. The only data from in vivo models have been obtained in genetically modified animals, i.e. ApoE- and LDL receptor deficient mice (LDLr-/-), respectively, with consequently high VLDL, LDL, and total Chl levels [6, 8]. In both models, *T*. *gondii* infection induced a decrease in total Chl and VLDL and LDL levels, attributed to acquisition by *T*. *gondii*, while HDL levels remained unaltered. However, these experiments were carried out in the setting of mice genetically modified to contain high levels of LDL.

But wild-type mice have very little LDL [9] and total Chl is largely composed of HDL [10]. We were thus interested in examining the influence of *T*. *gondii* infection on Chl metabolism in the normal host, and have shown that *T*. *gondii* infection indeed alters the lipid status in wild-type mice [11]. In an infection model in which changes in serum levels of Chl and its components were analyzed at weekly intervals post-infection (p.i.) until establishment of chronicity (d42 p.i.), the major changes observed in acute infection included a decrease in serum HDL, and accordingly total Chl, most significant on day 14 p.i.; as the infection converted into chronicity these changes were less prominent but persisted to d42 p.i. The decrease in host Chl reverse transport was attributed to its acquisition by *T*. *gondii*.

To further analyze the observed changes, namely, to explore whether the observed lipid metabolism alterations are genetically regulated, we proceeded to compare lipid metabolism in acute and chronic infection in serum and tissues including the brain and the liver, at both the pathophysiological and molecular level. The brain has been chosen as the target organ for encysted parasites, and the liver, although not a primary target of *T*. *gondii*, because of its central role in lipid metabolism. In addition, the observed gene expression of selected molecules involved in the Chl metabolic pathway was analyzed in the context of the results of published transcriptomes obtained in similar murine models of *T*. *gondii* infection.

## Material and methods

### Study design

The serum lipid status as well as the transcript levels of relevant genes in the brain and the liver have been assessed in experimental models of acute and chronic toxoplasmosis in wild-type mice. To put the obtained results into a wider context, we have mined all published microarray (n = 7) and RNA-seq (n = 1) datasets of murine tissues during acute infection with *T*. *gondii* type I, II and III strains [12, 13, 14], for the expression levels of genes relevant for Chl metabolism, including its biosynthetic pathway and export (KEGG pathways).

### Mice

For experimental infections, female Swiss-Webster mice (Medical Military Academy Animal Research Facility, Belgrade, Serbia) weighing 18–20 g, were used. Mice were housed at five per cage and offered regular mouse feed and drinking water ad libitum.

### Parasites

The low virulence BGD-1 strain (human origin type II strain) characterized in our laboratory [15] was used for experimental infections. Mice were inoculated by oropharyngeal gavage with 8 cysts, an inoculum shown not to be lethal for > 90% of the mice during the first eight weeks p.i. [15].

### Experimental design

To examine the serum lipid changes for each individual animal during *T*. *gondii* infection, groups of infected (n = 10) and control (n = 6) mice were euthanized at time of infection (d0), d14 p.i. and d42 p.i. At these points, blood samples were collected for measurement of lipid fractions, and livers and brains were harvested for real time-PCR analysis.

### Lipid measurements

Total Chl, HDL, LDL and triglyceride levels were measured on an Olympus AU 400 biochemical analyzer (Tokyo, Japan), according to the manufacturer’s recommendations.

### RNA extraction and cDNA synthesis

RNA was extracted from brain and liver tissue with TRIzol reagent (Invitrogen, Carlsbad, CA, USA) according to the manufacturer's protocol and the products were stored at −80°C until use. Total amount and RNA purity were determined on the Eppendorf ECOM-P4153 spectrophotometer (Hamburg, Germany) by absorbance at 260 nm and the 260/280 nm absorbance-ratio, respectively. Total cDNA was produced using the RevertAid First Strand cDNA Synthesis Kit (Fermentas, Vilnius, Latvia), according to the manufacturer's protocol. The cDNA products were stored −20°C until real time-PCR amplification.

### Real time-PCR screening

Relative quantification of the transcript levels of the selected genes (Table 1) was investigated using real-time PCR. Glyceraldehyde 3-phosphate dehydrogenase (Gapdh) gene was used as the reference one for gene expression analysis. Amounts of 50 ng of cDNA were subjected to real time-PCR amplification in 20 μl reactions that contained 1X Luminaris HiGreen qPCR Master Mix (Thermo Scientific, Wilmington, DE, USA) and 250 nM of each specific primer (Table 1). Intron spanning primer pairs were designed using NCBI Primer-BLAST tool and their specificity was confirmed through search against the human and *T*. *gondii* genomes and RefSeq mRNA databases. Amplicon specificity was verified by gel electrophoresis and melting curve analysis of qPCR products (data not shown). The real-time PCR amplifications were carried out in a Mastercycler ep realplex (Eppendorf, Hamburg, Germany) PCR machine using the following thermal profile: 10 min at 95°C, followed by 40 cycles of denaturation for 15 sec at 95°C, annealing and extension for 30 sec at 60°C (63°C for ApoA2). To avoid pipetting errors and to ensure that equal amount of cDNA was distributed across all PCR tubes for target and reference genes for each of the three biological replicate samples, cDNA was added to the master mix and mixed thoroughly before it was aliquoted into tubes already containing primer pairs. To control for DNA contamination in the reaction mix, control tubes lacking DNA templates were included, with primers for Gapdh for each biological replicate master mix. The Calqplex algorithm of the realplex software was used to determine the amplification cycle at which product accumulation was above the threshold (Ct).

**Table 1:** List of real time-PCR primers.

Gene	Real time-PCR forward primer	Real time-PCR reverse primer
Abca1	CTCAGAGGTGGCTCTGATGAC	CCCATACAGCAAGAGCAGAAG
ApoA1	GCACGTATGGCAGCAAGATG	GATTCAGGTTCAGCTGTTGGC
ApoA2	GCCTGGAAGGAGCTTTGGTTAAGA	TGCTGACCTGACAAGGGGTG
ApoB100	CGTGGGCTCCAGCATTCTA	AGTCATTTCTGCCTTTGCGTC
ApoC4	CTTGGTCAGCTTTGTAGCATCC	GGTCTGCATAAAGCCCTGGA
ApoE	CACACAAGAACTGACGGCAC	CGTAGATCCTCCATGTCGGC
ApoF	CGGACTGTATGGGTGCTCAG	CTGGTATCCCAACTTCGAGGG
Hmgcr	TCTGGCAGTCAGTGGGAACTATT	CCTCGTCCTTCGATCCAATTT
Ldlr	GGATCCACCGCAACATCTAC	TCTTTACGCCCTTGGTGTCA
SRBI	ACTTTTACAACGCCGACCCT	TCTTCCCTGTTTGCCCGATG
ApoC4	CTTGGTCAGCTTTGTAGCATCC	GGTCTGCATAAAGCCCTGGA
Gapdh	TGCCCCCATGTTTGTGATG	TGTGGTCATGAGCCCTTCC

### Statistical analysis

Data were analyzed using Gnumeric Spreadsheet 1.10.17 software and SPSS for Windows, version 16.0 (SPSS Inc, Chicago, IL, USA). Real-time PCR Ct values were analyzed using the 2−ΔΔCt method, where ΔΔCt = ΔCt samples– ΔCt controls. For each biological replicate, the Ct for a given gene was normalized to the Ct value of the reference Gapdh gene. Range of fold change expression was calculated by incorporating standard deviations (SD) of three biological replicates, from 2−(ΔΔCt−SD) to 2−(ΔΔCt+SD). Unpaired t-test analysis or Mann-Whitney U test were used to examine the differences in various variables between control and infected animals. P-values of 0.05 or less were considered statistically significant.

### Data mining

We mined data obtained from previously published transcriptomics studies in the model of experimental *T*. *gondii* infection. Literature search revealed three relevant studies, of which two were based on microarray [12, 13] and one on RNA-seq analysis [14].

The study of Jia B et al. from 2013 [12] involved expression profiling of brain and peripheral lymphocytes of mice infected with the RH (type I) or ME49 (type II) strains of *T*. *gondii*. Briefly, Balb/C mice were intraperitoneally (i.p.) infected with 10 tachyzoites per mouse, and brain tissue and peripheral lymphocytes were harvested 8 days p.i. for total RNA isolation. The gene expression profiling was done using Agilent Whole Mouse Genome (4 × 44 K) Microarrays (one-color platform). For the purpose of our study, we used four datasets of microarray hybridization results of up- and down- regulated genes in a) mouse brain after infection with parasites of the ME49 strain of *T*. *gondii*; b) mouse peripheral lymphocytes after infection with *T*. *gondii* parasites of the ME49 strain; c) mouse brain after infection by the *T*. *gondii* RH strain; and d) mouse peripheral lymphocytes after infection by the *T*. *gondii* RH strain.

The study of Hill et al. from 2012 [13] involved expression profiling of mouse peritoneal cells in mice infected with *T*. *gondii* strains GT1 (type I), PTG (type II), and CTG (type III). Briefly, CD-1 mice were infected intraperitoneally (i.p.) with 500 tachyzoites and total RNA from peritoneal cells was extracted 5 days p.i. Gene expression profiling was done using Affymetrix Mouse 1.0 ST array containing 28,853 genes. For our study we used data sets originating from infection with all three *T*. *gondii* strains.

The study of He et al. from 2016 [14] involved RNA-seq analysis of livers of Balb/C mice inoculated i.p. with 200 tachyzoites of the *T*. *gondii* PYS (Toxo DB#9) strain, which is of the I/III type. Mice were sacrificed 6 days p.i. and extracted RNA samples from liver were analysed using Illumina HiSeq 2000 system. Results were expressed as fold-change of differentially expressed genes in infected and uninfected mice for three biological replicates. We used these data to calculate averages of ratios of RPKM values. Ratio values between 0 and 1 were taken to indicate down regulation, while values above 1 were considered as up regulation.

### Ethics statement

The study protocol was approved by a local (Institute for Medical Research) Ethics Committee, approval no. 0091-2/10, and the State Ethics Committee (Veterinary Directorate of the Ministry of Agriculture and Environmental Protection of Serbia decision no. 323-07-02446/2014-05/1). All experiments were conducted concordant to procedures described in the National Institutes of Health Guide for Care and Use of Laboratory Animals (Washington, DC, USA).

## Results

Chl metabolism was analyzed in a murine model of *T*. *gondii* infection at two different time points: d14 p.i. and d42 p.i., as time points respectively representing acute and chronic infection. Chl metabolism was assessed at the pathophysiological and molecular levels, i.e. by measuring Chl content in the serum, and by examining the expression of selected genes involved in the regulation of Chl metabolism, respectively. The analyzed genes were selected based on their role and position in the physiological and metabolic pathways, in such a manner to have representatives at all critical points, while bearing in mind the cost-effectiveness of the experiment.

### Chl in serum and tissues in the acute infection model

**Fig 1:**
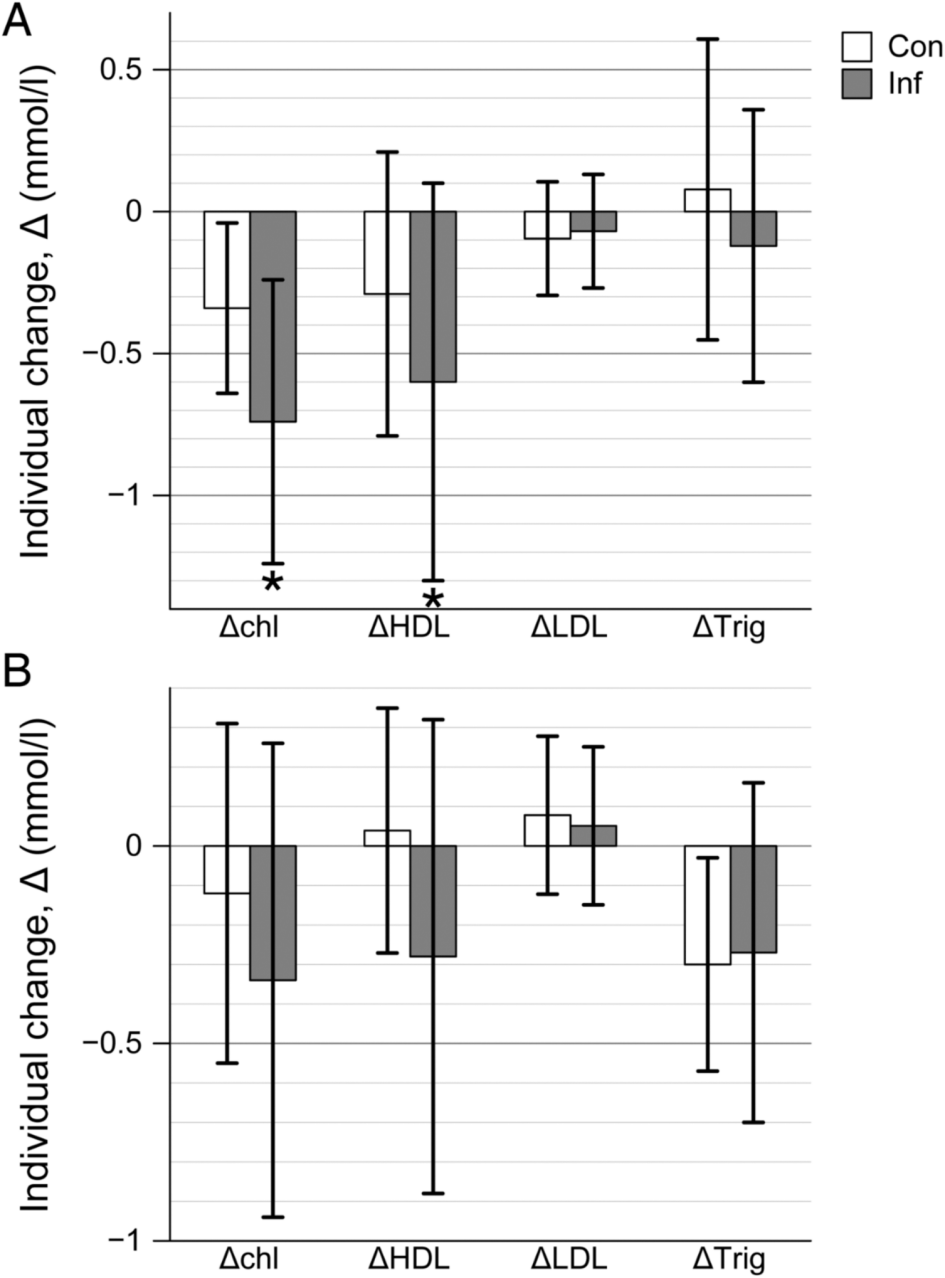
Changes in cholesterol (ΔChl), HDL (ΔHDL), LDL (ΔLDL), and triglycerides (ΔTrig) in *T*. *gondii* infection in mice (results presented as mean value of individual changes in groups of *T*. *gondii* infected and uninfected (control) mice). Panel A–acute toxoplasmosis; Panel B–chronic toxoplasmosis. * p<0.05 compared to control.

Analysis of individual changes in serum lipids on d14 p. i. revealed some significant changes in the lipid fractions as compared to pre-infection levels (Fig 1A). Both total Chl and HDL were significantly lowered on d14 compared to the time of infection in infected compared to control mice (-0.74 ±0.5 vs. -0.34 ± 0.3 and -0.6 ± 0.7 vs. -0.29 ± 0.5; p<0.05 respectively), while there was no such difference in the change of serum LDL and triglycerides (-0.06 ± 0.20 vs. -0.09 ± 0.2) and -0.12±0.48 vs. 0.08 ± 0.53; p>0.05, respectively).

Expression analysis of the selected genes involved in Chl metabolism showed that acute infection was associated with a decrease in the transcription of genes involved in Chl biosynthesis in both the liver and the brain.

**Fig 2:**
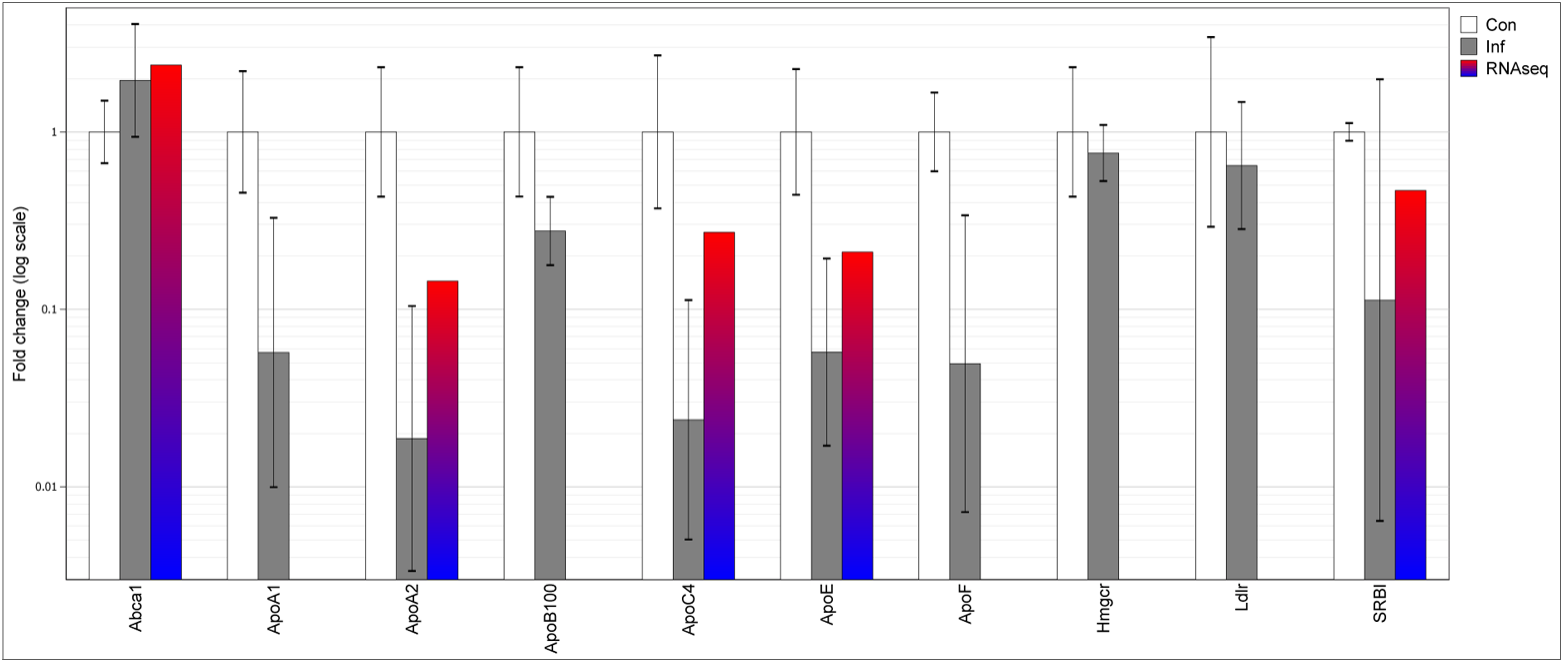
Hepatic mRNA expression of genes involved in lipid homeostasis in acute *T*. *gondii* infection, determined by qPCR and RNA-seq. Original RNA-seq data is from He et al. [14]. Differential gene expression is represented by fold change values normalized to control samples. Error bars represent standard deviations of the ΔΔCt values incorporated into the fold change. Con–uninfected control; Inf–infection; *–p<0.05 compared to control.

In the liver, acute *T*. *gondii* infection was associated with a significant increase in the expression of Abca1 gene. In contrast, there was a decrease in the expression of all other analyzed genes, including Ldlr and SR-B1 genes. Significant down-regulation was observed in the expression of apolipoprotein genes including ApoA1, ApoA2, ApoB100, ApoC4 and ApoE (Fig 2), and a decrease of borderline significance was observed in the expression of ApoF expression (P = 0.053).

**Fig 3:**
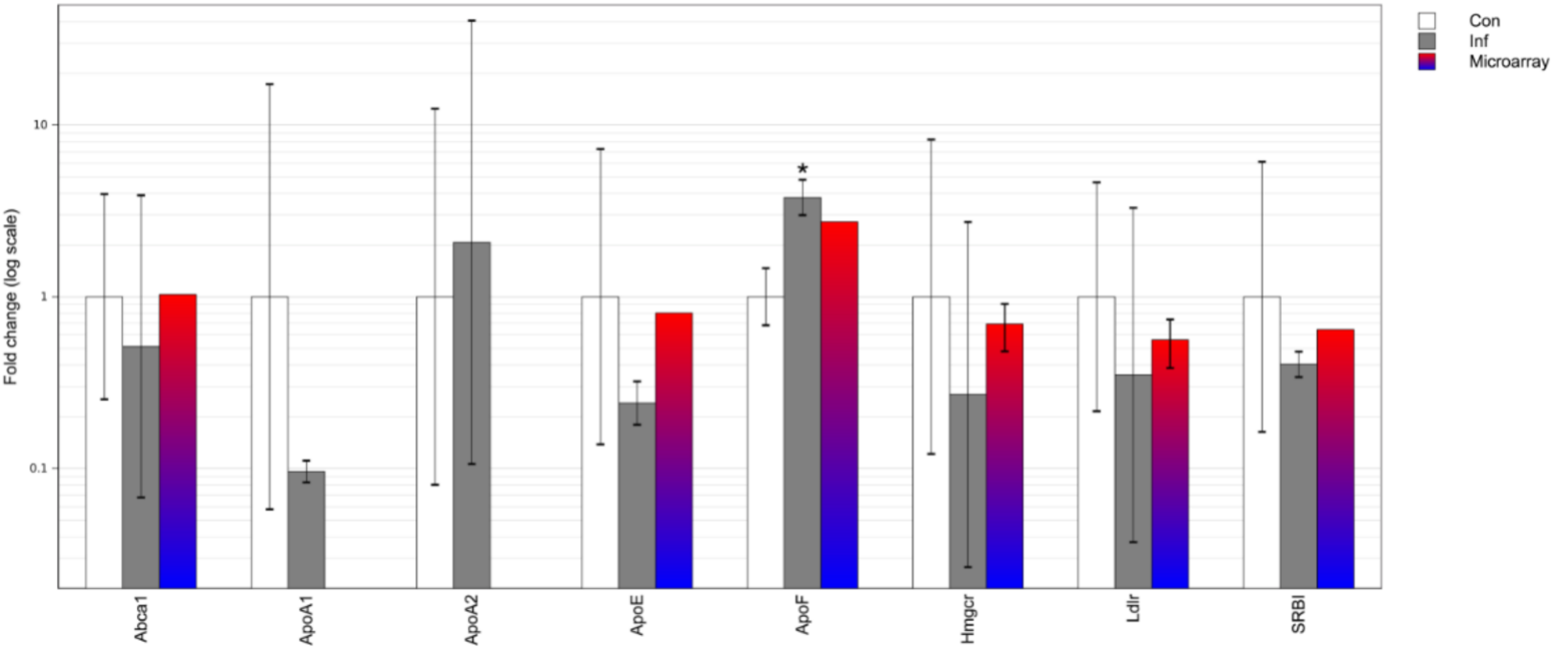
Brain mRNA expression of genes involved in lipid homeostasis in acute *T*. *gondii* infection, determined by qPCR and microarray. Differential gene expression is represented by fold change values normalized to control samples. Error bars represent standard deviations of the ΔΔCt values incorporated into the fold change for qPCR data or standard deviations of the microarray probes’ fold changes for genes covered with multiple microarray probes. Original genome-wide microarray data is from Jia B et al. [12]. Con–uninfected control; Inf–infection; *–p<0.05 compared to control.

In the brain, a highly pronounced up-regulation in ApoF expression was detected by real-time PCR analysis (Fig 3). Conversely, we observed a trend of down-regulation of ApoE and Hmgcr. Importantly, data mining showed that our observations matched those obtained by microarray analysis of mouse brain in acute (8d) *T*. *gondii* type II infection (Fig 3), where there was also up-regulation of ApoF and down-regulation of ApoE and Hmgcr.

### Chl in serum and tissues in the chronic infection model

In chronic infection (d42 p.i.), few changes vs. control mice were registered in the serum (Fig 1B). No differences in the change of any of the lipid fractions (total serum Chl, HDL, LDL, and triglycerides) vs. pre-infection values were noted between infected and control mice (p>0.05), which means that an increase back to normal values in Chl metabolism had occurred.

**Fig 4:**
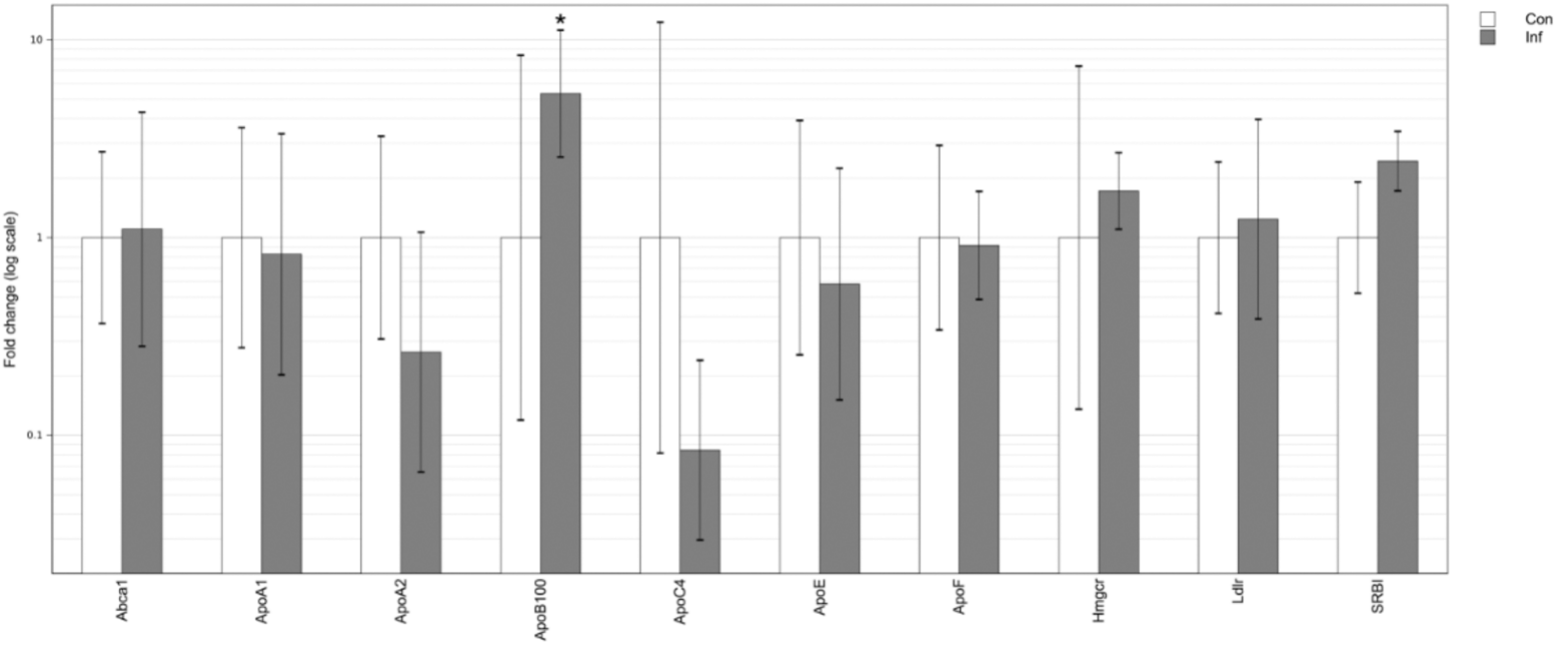
Hepatic mRNA expression of genes involved in lipid homeostasis in chronic *T*. *gondii* infection. Differential gene expression is represented by fold change values normalized to control samples. Error bars represent standard deviations of the ΔΔCt values incorporated into the fold change. Con–uninfected control; Inf–infection; *–p<0.05 compared to control.

At the gene level, chronic infection in the liver was associated with a significant increase in the expression of only ApoB100 (Fig 4). The expression of all other analyzed genes did not differ significantly compared to control mice, lending support at the molecular level to the absence of differences seen in the serum.

**Fig 5:**
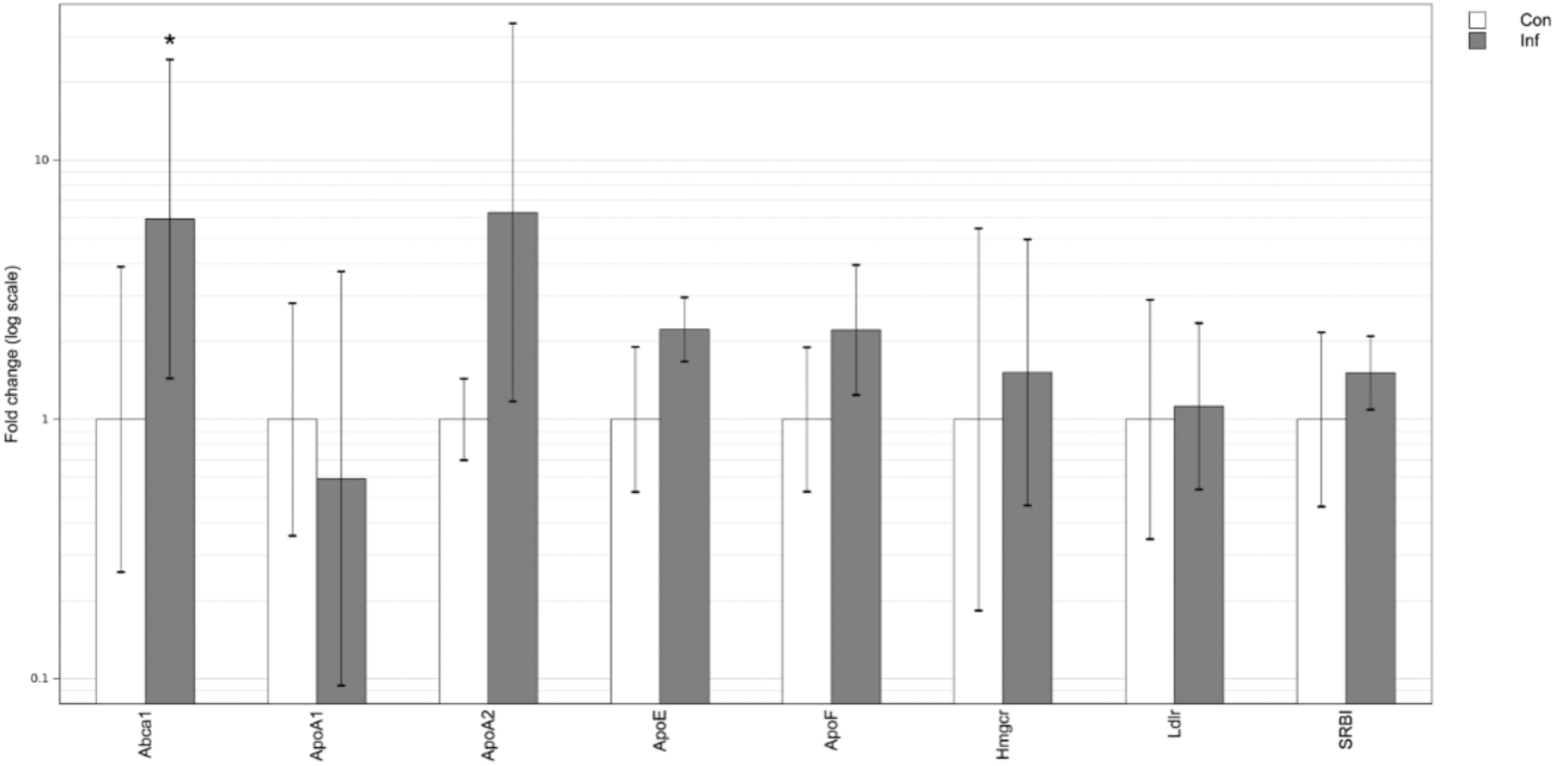
Brain mRNA expression of genes involved in lipid homeostasis in chronic *T*. *gondii* infection. Differential gene expression is represented by fold change values normalized to control samples. Error bars represent standard deviations of the ΔΔCt values incorporated into the fold change. Con–uninfected control; Inf–infection; *–p<0.05 compared to control.

In the brain, significant up-regulation in the expression of only Abca1 was observed, along with a trend of up-regulation of genes including ApoA2, ApoE, ApoF, Hmgcr and SRB-I (Fig 5).

### Data mining

**Fig 6:**
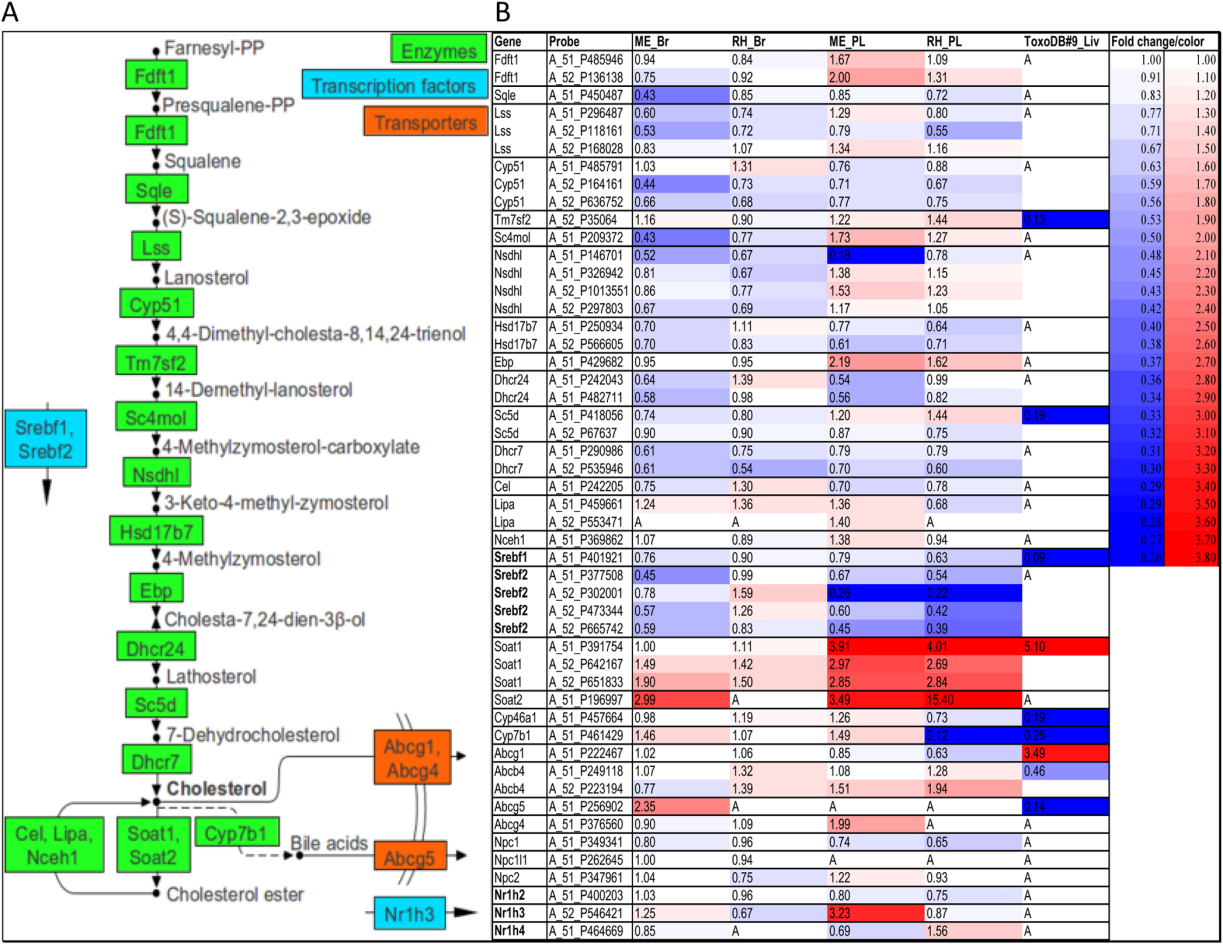
Transcriptome expression profiles of selected genes involved in cholesterol biosynthesis and export in acute *T*. *gondii* infection. Microarray data in brain and peripheral lymphocytes in infection with ME49 (type II) and RH (type I) parasites (data from Jia et al. [12]). RNa-seq data in liver in infection with PYS (type I/III) parasites (data from He et al. [14]). Panel A–extract from cholesterol biosynthetic pathway and export (adapted from KEGG pathways). Panel B–differential gene expression represented by fold change values / color for each microarray probe or gene: mouse brain in *T*. *gondii* type II / type I infection (ME_Br / RH_Br); mouse peripheral lymphocytes in *T*. *gondii* type II / type I infection (ME_PL / RH_PL); mouse liver in *T*. *gondii* strain PYS (type I/III). A–not identified as a differentially expressed gene in all three biological replicates.

A compilation of all published transcriptome data obtained in murine toxoplasmosis pertaining to Chl metabolism is presented in Fig 6.

In the brain of mice infected with type II parasites in acute infection (day 8 p.i.), the data revealed down-regulation of most of the 13 genes from the Chl biosynthetic pathway starting from farnesyl-PP, and of two transcriptional activators of this pathway, Srebf1 and Srebf2 (Fig 6). On the other hand, Soat1 and Soat2, as well as Cyp7b1 and Abcg5 were upregulated. While up-regulation of Soat1 and Soat2 reflects an increase in Chl esterification, up-regulation of Cyp7b1, which in extrahepatic tissues catalyzes the first reaction in the Chl catabolic pathway towards bile acids, and of bile acid export protein Abcg5, reflect removal of Chl from a metabolically active pool. The expression profile of these genes in murine brain during infection with type I parasites (d5 p.i.) was similar but less pronounced when compared to type II infection.

Peripheral lymphocytes seemed to show a similar expression profile. Down-regulation of several genes from the Chl biosynthesis pathway and up-regulation of genes involved in its esterification were observed during infection with both type I and type II parasites. Furthermore, in infection with type II parasites, up-regulation of Abcg4, a Chl export protein, and of liver X receptor alpha (Nr1h3), was revealed.

In the liver of mice in acute *T*. *gondii* infection (day 6 p.i.), several genes from the Chl biosynthetic pathway were down-regulated. Furthermore, Cyp7b1 and Abcg5, involved in bile acid synthesis and export, were down-regulated. On the other hand, Abcg1, involved in Chl export, and Soat 1, involved in Chl esterification, were up-regulated.

In peritoneal cells of mice infected with *T*. *gondii* type I, II and III parasites, mining for differentially expressed genes involved in Chl biosynthesis and transport revealed an over two-fold increase in several genes, including Hmgcr, Fdft1, Sqle and Ldlr, while the expression of ApoE was reduced by more than six-fold (data not shown).

Integration of our experimental data with those obtained by mining is schematically represented in Table 2. The main observation in our experiments was a decrease in total serum Chl and HDL levels in acute infection, while both returned to control values in chronic infection. At the molecular level, all presented results show that in acute toxoplasmosis (data from four different experiments on d5, 6, 8 and 14 p.i.) there was a decrease in the level of transcription of genes involved in Chl biosynthesis in all organs except in peritoneal cells, where an increase in three genes from this pathway (Hmgcr, Fdft1 and Sqle) was observed. The chronic stage of *T*. *gondii* infection (day 42 p.i.) was characterized by a return back to normal values of Chl metabolism, which was manifested at the molecular level by few differences, including upregulation of ApoB100 in the liver and of Abca1 in the brain.

**Table 2:** Scheme of time—Dependent and organ—Specific changes in cholesterol metabolism during *T*. *gondii* infection. Decrease and increase in infected vs. uninfected mice.

	Tissue				
Infection (days)	Serum	Liver	Brain	Lymphocytes	Peritoneal cells
Acute (5, 6, 8,14 days)	↓	↓	↓	↓	↑
Chronic (42 days)	↔	↔	↔	x	x

↓ - decreased cholesterol; ↑ - increased cholesterol; ↔ - unchanged cholesterol; x–no data.

## Discussion

In this study, we analyzed Chl metabolism during acute and chronic *T*. *gondii* infection in murine models induced by type II parasites at both the biological and molecular level. Combining own experimental data with those mined from published transcriptomes of *T*. *gondii* infected mice, we showed that acute infection was associated with a decrease in Chl content in both the liver and periphery (brain, peripheral lymphocytes), and a decrease in Chl reverse transport. In contrast, in chronic infection, at the time of an established balance between the parasite and its host, these metabolic changes were not as prominent.

We have specifically analyzed the liver and the brain. In the liver, acute *T*. *gondii* infection was associated with a significant increase in the expression of Abca1 and with down-regulation (or a trend of) of a number of apolipoprotein genes as well as of the Ldlr and SR-B1 genes. Since Abca1 plays a key role in hepatic Chl efflux [16], its increased gene expression may point to increased removal of Chl from the hepatocytes resulting in its reduced availability to proliferating tachyzoites. On the other hand, decreased expression of various apolipoproteins has been observed (with or without decreased plasma levels) in different infection settings, such as sepsis, acute bacterial and fungal infection, and HIV infection [17, 18]. In addition, decreased expression of SR-B1 may be important as a mechanism to regulate Chl uptake by hepatocytes. Moreover, it may be speculated that the decrease of SR-B1 expression, which in turn reduces Chl intake by hepatocytes, may also limit entry of *T*. *gondii* into hepatocytes, as has been shown for the closely related Apicomplexan *Plasmodium* sporozoites [19]. Taken together, the net effect of these processes is decrease of Chl content in the liver, which is exactly what was observed in both experimental infection and by data mining [14].

In the brain during acute infection, we observed a reduction in Chl levels. At the level of transcription, significant up-regulation of ApoF was observed, along with a trend of down-regulation of a number of analyzed genes involved in Chl intake, trafficking and biosynthesis. Normally, ApoF forms complexes with lipoproteins and may be involved in transport and/or esterification of Chl and has also been shown to act as a stimulator of Chl transport from HDL to VLDL and LDL, causing a decrease in serum level of HDL [20]. On the other hand, down-regulation of the LDLr gene suggests a decrease in the uptake of Chl by the brain. Data mining results in a similar model showed decreased expression of transcription factors Srebf1 and Srebf2 and of enzymes involved in Chl synthesis (Hmgcr, Cyp51 and others in the steroid biosynthetic pathway), but an increase in the transcription of the Soat1 and Soat2 enzymes which catalyze Chl esterification to Chl-palmitate, as well as of Abcg5, pointing to an increased Chl ester efflux from the brain. Similarly, as in the liver, all these processes lead to a reduction in the brain Chl content. This has indeed been actually observed in experimentally infected mice.

The reduction of brain Chl may be part of the host defensive reaction to the parasite. The brain is the major site of parasite replication and development [21]. We have previously shown in a similar infection model that there may be more than 10,000 parasites per mL of tissue in the brain by d14 [22], a significant burden which induces a strong host defensive response. The decrease in the Chl content may be aimed at decreasing the rate of parasite replication, and/or possibly triggering tachyzoite-bradyzoite conversion. This decrease is concomitant with the decrease in serum levels of HDL, confirming the reduction of reverse Chl transport to the liver, which we showed previously [11]. Further evidence to support the decrease in reverse Chl transport is presented by increased expression of ApoF.

Analysis of the published transcriptomes showed significant changes in the lymphocytes as well. We did not verify this in our experimental model, but it is interesting to note that mining showed a decrease in the level of transcription of genes involved in Chl biosynthesis, and up-regulation of genes involved in its esterification, as well as an increased level of liver X receptor alpha, which may all lead to a decrease in Chl accumulation and increased Chl efflux from the lymphocytes. Chl content in the plasma membrane of immune cells causes alteration in membrane viscosity, influencing functions such as maturation [23], antigen presentation [24], activation [25, 26], and killing by NK cells [27]. Moreover, lymphocyte activation during acute *T*. *gondii* infection may contribute *per se* to impaired lipid metabolism and decreased Chl content.

At the time of established chronicity (d42 p.i.), a return to normal levels of Chl metabolism has been noted. Total serum Chl and HDL levels, both decreased in acute infection, were both comparable to controls in chronic infection. This has also been supported by fewer differential gene expression findings at this time; the only upregulated gene in the liver was ApoB100, which may suggest increased transport of LDL from the liver to the periphery. More importantly, changes in the brain on day 42 included increased Abca1 gene expression along with (a trend of) upregulation of ApoE. Neurons have been shown to be the primary target cells of *T*. *gondii* in the brain [28]. However, astrocytes, and not neurons, are the main brain cell source of Chl. Chl needs of the neurons are supplied by astrocytes, from which Chl is exported by Abca1 and transported by ApoE [29]. Accordingly, increased expression of Abca1 and ApoE detected in *T*. *gondii* infected mice on d42 p.i. supports increased export and transport of Chl to the neurons, possibly in response to increased Chl needs of parasitized neurons due to developing cysts, and/or as a neuron repair mechanism. Increase in Chl metabolism components is the normal brain response to injury or disease [29]. It would be interesting to examine these same molecules later during the course of *T*. *gondii* infection, when the infection is patent, as compared to d42 which is an early moment of established chronicity, still associated with cyst development and growth. In addition, the impact of chronic infection on lipid metabolism should be examined in other cyst predilection sites such as the heart and skeleton muscles.

In its proliferative stage, the parasite needs Chl for development. It has been shown that Chl is uptaken in the form of LDL, through the Ldlr [6]. However, in wild-type mice the fraction of LDL within the composition of normal Chl is limited. It is possible that the increased uptake of LDL could not be seen at the serum level in our model because of the miniscule amounts of LDL in the normal Chl composition in rodents, but the decrease in its reverse transport to the liver in the form of HDL indirectly indicates LDL has been used up in the periphery. On the other hand, it may be that the parasite uptakes Chl in any form available to it, which in mice is HDL. This may be supported by the decrease in HDL levels seen in acute infection associated with the decrease in the HDL receptor SR-B1 gene expression, which suggests it may have been taken up in the periphery for the parasite needs by some alternative pathway. Mice infected with parasites such as *Trypanosoma cruzi* and *Schistosoma mansoni* were found to have decreased levels of total blood Chl. In the trypanosome infection model, Chl fractions have not been analyzed [30]. On the other hand, although *S*. *mansoni* is a trematode completely unrelated to *Toxoplasma*, schistosomes do not synthesize Chl but incorporate host LDL via inducible LDL receptors. In *S*. *mansoni* infected outbred mice, a reduction in total Chl and HDL and an insignificant rise in LDL was shown at a time at which infection became patent [31]. In contrast, infection with, for instance, the intracellular bacterium *Chlamydia pneumoniae* has been shown to cause significant changes in liver lipid metabolism, but, as opposed to the above organisms, which result in increased serum Chl and triglyceride levels [32, 33].

Limitations to our study and the presented results include variations in the experimental models between ours and those in the mined data complicating direct comparisons; the model used by Jia et al. [12] is largely comparable to ours (*T*. *gondii* infecting strain, route of infection) but they used inbred mice and collected the samples d8 p.i. as opposed to d14 in our model. On the other hand, the model used by He et al. [14] also used outbred mice but differs in the *T*. *gondii* strain (type I/III as opposed to type II in our model) and in the route of infection as they used the intraperitoneal route. A limitation intrinsic to our experimental model involves the variations among individual animals obtained both at the physiological and molecular level, as visualized by occasionally wide standard deviations, the reason for which is likely to be our use of outbred mice for experimental infections. Wild-type mice have been shown to widely differ in their lipid content [30]. It is possible that the use of inbred animals would have afforded more homogeneous results. However, the mere fact that we did obtain statistically significant differences in some parameters and not in others even with mice with wide initial individual variations lends additional weight to the results themselves, emphasizing their true value.

We propose that the observed changes in Chl metabolism are part of the host defense response. In acute infection, the host responds by an attempt to deprive the parasite of Chl, necessary for tachyzoite proliferation and development. The influence of *T*. *gondii* infection on Chl metabolism may have a significance beyond this immediate effect, as Chl influences various key physiological processes such as role in the metabolism of lipid soluble vitamins, synthesis of sex hormones etc.

The variety of lipid metabolism alterations induced by *T*. *gondii* infection has opened exciting new therapeutic approaches. Coppens et al. [3] have proposed that the dependence of *T*. *gondii* on exogenous Chl may be used for the delivery to the parasite of lipid analogues with parasitostatic effect. It is hoped that further insight may lead to additional novel targets for therapeutic agents.
